# Effects of ozone therapy on anxiety and depression in patients with refractory symptoms of severe diseases: a pilot study

**DOI:** 10.3389/fpsyg.2023.1176204

**Published:** 2023-08-04

**Authors:** Bernardino Clavo, Angeles Cánovas-Molina, Juan A. Díaz-Garrido, Silvia Cañas, Yolanda Ramallo-Fariña, Horus Laffite, Mario Federico, Delvys Rodríguez-Abreu, Saray Galván, Carla García-Lourve, Damián González-Beltrán, Miguel A. Caramés, Jose L. Hernández-Fleta, Pedro Serrano-Aguilar, Francisco Rodríguez-Esparragón

**Affiliations:** ^1^Research Unit, Hospital Universitario de Gran Canaria Dr. Negrín, Las Palmas de Gran Canaria, Spain; ^2^Chronic Pain Unit, Hospital Universitario de Gran Canaria Dr. Negrín, Las Palmas de Gran Canaria, Spain; ^3^Radiation Oncology Department, Hospital Universitario de Gran Canaria Dr. Negrín, Las Palmas de Gran Canaria, Spain; ^4^Fundación Canaria Instituto de Investigación Sanitaria de Canarias (FIISC), Las Palmas de Gran Canaria/Tenerife, Spain; ^5^Universitary Institute for Research in Biomedicine and Health (iUIBS), Molecular and Translational Pharmacology Group, University of Las Palmas de Gran Canaria, Las Palmas de Gran Canaria, Spain; ^6^Instituto Universitario de Enfermedades Tropicales y Salud Pública de Canarias de la Universidad de La Laguna, Santa Cruz de Tenerife, Spain; ^7^CIBER de Enfermedades Infecciosas (CIBERINFEC), Instituto de Salud Carlos III, Madrid, Spain; ^8^Spanish Group of Clinical Research in Radiation Oncology (GICOR), Madrid, Spain; ^9^Psychiatry Department, Hospital Universitario de Gran Canaria Dr. Negrín, Las Palmas de Gran Canaria, Spain; ^10^Psychiatry Department, Complejo Hospitalario Universitario Insular Materno-Infantil de Gran Canaria, Las Palmas de Gran Canaria, Spain; ^11^Network for Research on Chronicity, Primary Care, and Health Promotion (RICAPPS), Santa Cruz de Tenerife, Spain; ^12^Servicio de Evaluación y Planificación del Servicio Canario de Salud (SESCS), Santa Cruz de Tenerife, Spain; ^13^Instituto de Tecnologías Biomédicas (ITB), Universidad de la Laguna, Santa Cruz de Tenerife, Spain; ^14^Medical Oncology Department, Complejo Hospitalario Universitario Insular Materno-Infantil de Gran Canaria, Las Palmas de Gran Canaria, Spain; ^15^Medical Oncology Department, Hospital Universitario de Gran Canaria Dr. Negrín, Las Palmas de Gran Canaria, Spain

**Keywords:** ozone therapy, anxiety and depression, health-related quality of life, advanced diseases, chemotherapy-induced side effects, radiation-induced side effects, cancer survivors, chemotherapy-induced neuropathy

## Abstract

**Background:**

Patients with refractory symptoms of severe diseases frequently experience anxiety, depression, and an altered health-related quality of life (HRQOL). Some publications have described the beneficial effect of ozone therapy on several symptoms of this kind of patient. The aim of this study was to preliminarily evaluate, in patients treated because of refractory symptoms of cancer treatment and advanced nononcologic diseases, if ozone therapy has an additional impact on self-reported anxiety and depression.

**Methods:**

Before and after ozone treatment, we assessed (i) anxiety and depression according to the Hospital Anxiety and Depression Scale (HADS); (ii) the HRQOL (according to the EQ-5D-5L questionnaire), which includes a dimension on anxiety and depression and a visual analog scale (VAS) measuring self-perceived general health.

**Results:**

Before ozone therapy, 56% of patients were on anxiolytic and/or antidepressant treatment. Before and after ozone therapy, the anxiety and depression HADS subscales (i) significantly correlated with the anxiety/depression dimension of the EQ-5D-5L questionnaire and (ii) inversely correlated with the health status as measured by the VAS. After ozone therapy, we found a significant improvement in anxiety and depression measured by both the (i) HADS subscales and (ii) EQ-5D-5L questionnaire.

**Conclusion:**

The addition of ozone therapy for patients with refractory symptoms of cancer treatment and advanced chronic nononcologic diseases can decrease anxiety and depression severity levels. Additional, more focused studies are ongoing to provide the needed explanatory information for this finding.

## 1. Introduction

Patients with refractory symptoms of cancer treatment and advanced nononcologic diseases frequently experience anxiety, depression, and an altered health-related quality of life (HRQOL). Fortunately, the improvements in diagnostics and treatments over the last decades have increased survival rates in patients with cancer or advanced chronic diseases. In the European Union (EU), the relative average number of cancer survivors was more than 50% within 5 years of diagnosis during 2000–2007 in both sexes (European Commission, 2000) and the prevalence of depression is approximately 20% in cancer survivors (Boyes et al., 2013). In addition, the general population frequently reports chronic anxiety and depression (7.2%) (European Commission, 2023), with increased levels in patients with advanced chronic disease (DeJean et al., 2013; Li et al., 2019) or cancer (Greer et al., 2011; Gotze et al., 2020; Ji et al., 2020). However, in these patients, anxiety and depression can be associated with an increased risk of all-cause mortality (Lloyd et al., 2019), which relevantly impacts the results of symptom management and their HRQOL.

Anxiety and depression are associated with higher levels of oxidative stress markers and pro-inflammatory cytokines as well as with decreased levels of antioxidants (Ng et al., 2008; Maes et al., 2011; Leonard and Maes, 2012; Lindqvist et al., 2017). Furthermore, the success of antidepressant treatment is associated with changes in those parameters over the course of treatment (Lindqvist et al., 2017). Interestingly, most of the scientific reports about ozone and anxiety and depression have been focused on “ozone inhalation” to induce oxidative stress as a mechanism of production of anxiety and depression (Gonzalez-Pina and Paz, 1997; Avila-Costa et al., 1999; Santiago-Lopez et al., 2010; Mokoena et al., 2015).

However, (i) medical ozone treatment (O_3_T) must specifically avoid the inhalation of ozone, and (ii) an appropriate ozone concentration and route of administration look for the induction of a controlled, limited and transient oxidative stress that can overregulate nuclear factor erythroid 2-related factor 2 (Nrf2), which is the key for a further enhancement of the antioxidant defenses in the body with further and favorable modulation of oxidative stress and inflammation (Re et al., 2014; Bocci and Valacchi, 2015; Galie et al., 2019; Viebahn-Haensler and Leon Fernandez, 2021).

In cancer survivors with refractory symptoms, adjuvant O_3_T can reduce several chronic symptoms such as ischemic and metabolic issues (Clavo et al., 2011), pelvic pain (Clavo et al., 2021), radiation-induced hematuria (Clavo et al., 2005) or hemorrhagic proctitis (Clavo et al., 2013, 2015), chemotherapy-induced peripheral neuropathy (Clavo et al., 2022), and lymphedema (Waked et al., 2013).

In a recent study of cancer survivors with chronic symptoms, we found that O_3_T could decrease the grade of toxicity secondary to cancer treatments as well as improve HRQOL using the EQ-5D-5L questionnaire, which includes an anxiety/depression dimension that also showed improvement after O_3_T (Clavo et al., 2023). However, the EQ-5D-5L questionnaire assesses anxiety and depression with only one question and it seemed justified the evaluation with a specific questionnaire focused on anxiety and depression.

The aim of this study was to assess (in patients treated because of refractory symptoms of severe diseases) if O_3_T has an additional impact on self-reported anxiety and depression using a specifically focused questionnaire: the Hospital Anxiety and Depression Scale (HADS) (Zigmond and Snaith, 1983).

## 2. Materials and methods

### 2.1. Patients

This is a retrospective case series of 16 patients submitted to our Chronic Pain Unit between November 2019 and October 2022, with these inclusion criteria: (i) they were treated with O_3_T because of chronic and refractory symptoms of severe diseases and (ii) they had completed HADS questionnaires before and after O_3_T. They were 13 cancer survivors with chronic side effects of cancer treatment (8 because of chemotherapy-induced peripheral neuropathy and 5 because of local toxicity induced by radiochemotherapy) and 3 noncancer patients with chronic symptoms of advanced diseases (because of cardiopathy, cerebellar syndrome, and post-COVID-19 syndrome). Informed written consent was obtained from all patients, according to the Declaration of Helsinki of 1975. The administration of O_3_T in our hospital was assessed by the Health Care Ethics Committee. This research study was approved by the Provincial Research Ethics Committee of Las Palmas, Spain (Ref 2019–288-1) on 2 December 2022. Table 1 shows patient’s clinical characteristics.

**Table 1:** Patient’s clinical characteristics.

#	Age, years	Sex	O3T	Anxio. Tx	Depre. Tx	Clinical characteristics
1	73	F	S	Yes	No	Uterine carcinoma: Surg. + CT + RT. O_3_T for CIPN. Tx: Bromazepam, Pregabalin
2	56	M	S	Yes	No	Ischemic cardiopathy grade III. O_3_T for physical fatigue. Tx: Diazepam
3	54	M	L*	No	No	Rectal Kaposi’s sarcoma: Surg. + CT + RT. O_3_T for wound dehiscence.
4	72	M	S	Yes	No	Unknown cerebellar syndrome. O_3_T for instability and movement disturbances. Tx: Clonazepam, Pregabalin
5	67	F	S + L	No	No	Uterine cervical carcinoma: RT + CT. O_3_T for radiation-induced hematuria.
6	45	F	S	No	No	Ovarian carcinoma + uterine carcinoma: Surg. + RT. O_3_T for CIPN.
7	69	M	S	No	No	Head and neck carcinoma + cutaneous melanoma: Surg. + CT. O_3_T for CIPN.
8	65	F	S + L	No	No	Relapsed vaginal carcinoma: RT + CT. O_3_T for vulvar and vaginal wounds.
9	62	M	S	Yes	Yes	Colon carcinoma: Surg. + CT. O_3_T for CIPN. Tx: Alprazolam
10	53	F	S	Yes	Yes	Non-Hodgkin lymphoma: Surg. + RT + CT. O_3_T for CIPN. Tx: Pregabalin, Amitriptyline, Citalopram
11	53	F	L*	Yes	Yes	Relapsed carcinoma of uterine cervix and vagina: RT + CT. O_3_T for vulvar and vaginal wounds. Tx: Pregabalin, Duloxetine
12	71	M	S	No	Yes	Pancoast carcinoma of the lung: CT + RT. O_3_T for CIPN + refractory post-herpetic neuralgia. Tx: Duloxetine
13	68	M	S	No	No	Pancoast carcinoma of the lung: CT + RT. O_3_T for CIPN.
14	76	F	S	Yes	No	Uterine carcinosarcoma: Surg. + CT + RT. O_3_T for CIPN. Tx: Alprazolam
15	69	F	L*	Yes	Yes	Rectum carcinoma: Surg. + CT + RT. O_3_T for wound dehiscence. Tx: Clonazepam, Venlafaxine
16	49	M	S	No	No	COVID-19 disease: Prolonged hospital stay. O_3_T for secondary polyneuropathy.

#: Patients. O_3_T, ozone therapy; S, systemic ozone therapy; L, local ozone therapy; S+ L, systemic and local ozone therapy; Tx, treatment; Anxio. Tx, anxiolytic treatment; Depre. Tx., antidepressant treatment; CIPN, chemotherapy-induced peripheral neuropathy. All symptoms treated with ozone therapy were chronic and refractory symptoms. Surg., surgery; CT, chemotherapy; RT, radiotherapy. * “Local ozone administration alone” was used only for superficial wounds or wound dehiscences with localized symptoms.

### 2.2. Ozone therapy

O_3_T was administered on an outpatient basis, always avoiding the inhalation of ozone by patients or by staff. Ozone (an O_3_/O_2_ mixture) was obtained from clinical-grade oxygen using two medical ozone generators (Ozonosan Alpha-plus®, Dr. Hänsler GmbH, Iffezheim, Germany; Ozonobaric P®, Sedecal, Madrid, Spain).

O_3_T was administered according to the symptoms of the patients. A total of 13 patients (81%) received systemic ozone treatment, 11 by rectal insufflation (1 with additional topical treatment), and 2 by autohemotherapy (1 with additional local treatment). Five patients (31%) received local O_3_T, three (19%) as an exclusive procedure, and two with additional systemic treatment. We have previously described the procedures followed for rectal insufflation (Clavo et al., 2013) and autohemotherapy (Clavo et al., 2013); the O_3_/O_2_ concentrations were progressively increased from 10 to 30 μg/mL and from 30 to 50 μg/mL, respectively. For topical administration, O_3_/O_2_ concentrations usually ranged between 10 and 40 μg/mL according to patient tolerance or based on the absence or presence of local infection. In cancer survivors, O_3_T was administered if evidence of tumor progression was lacking.

### 2.3. Anxiety and depression assessment

The aim of this study was the assessment of anxiety and depression using the Spanish version of the HADS (Zigmond and Snaith, 1983; Herrero et al., 2003; Quintana et al., 2003; Mitchell et al., 2010). The HADS questionnaire includes 14 questions, which can be scored from 0 (best) to 3 (worst). It includes seven questions assessing for anxiety (HADS-A) and seven questions assessing for depression (HADS-D). The maximum score for each subscale is 21: 0–7, normal; 8–10, mild; 11–15, moderate; 16–21, severe.

We also assessed the HRQOL with the Spanish version (v1.0, 2009) of the EQ-5D-5L questionnaire. Their cultural adaptation was carried out following the methodology recommended by the EuroQol Group (Rabin et al., 2014), and its validity for Spain and the United Kingdom has been demonstrated in different studies (Herdman et al., 2011; Hernandez et al., 2018; Ramos-Goni et al., 2018). The EQ-5D-5L assesses five different dimensions scored from 1 (“I have no problems”) to 5 (“I have a lot of problems”): (i) mobility, (ii) self-care, (iii) activities of daily living, (iv) pain and discomfort, and (v) anxiety/depression. The EQ-5D-5L also includes a visual analog scale (VAS) measuring self-perceived general health status (EQ VAS), scored from “0” (worst health status) to “100” (best health status).

### 2.4. Statistical analysis

The SPSS software package (version 15 for Windows) was used for statistical analyses. All data are described as median (quartile 2) and quartiles 1 and 3 (Q1–Q3). The correlation between the grade of toxicity and EQ-5D-5L dimensions was assessed with Spearman’s rho. Paired comparisons (before/after O_3_T) were conducted with the exact (significance) Wilcoxon rank test. Unpaired comparisons (before/after O_3_T) were conducted with the exact (significance) Mann–Whitney *U*-test. Qualitative variables were compared with the exact (significance) McNemar’s test. Though more conservative than asymptotic tests, exact tests were used due to the small sample size. *p*-values of <0.05 were considered statistically significant.

## 3. Results

The sex distribution included eight men and eight women. The median age was 66 years (Q1–Q3 = 53.3–70.5). Symptoms treated with O_3_T were previously present for a median of 14.5 months (Q1–Q3 = 8.3–20). Overall, the median number of systemic O_3_T sessions was 40 (Q1–Q3 = 40–40), and the number of local O_3_T sessions was 40 (Q1–Q3 = 25–61.5). The median duration of O_3_T was 20 weeks (Q1–Q3 = 17–25).

Before the commencement of O_3_T, nine patients (56%) were taking anxiolytics (eight patients) or antidepressants (five patients), with four patients taking both therapies. Patients were treated with O_3_T because of chronic and refractory symptoms and treatment for anxiety and depression was not prescribed nor modified in our Chronic Pain Unit during O_3_T.

Anxiety assessed by the HADS-A did not show significant differences between the patients without or with anxiolytic treatment (i) before O_3_T, 4.5 (Q1–Q3 = 3–9.5) vs. 9.5 (Q1–Q3 = 6–13), *p* = 0.207 or (ii) after O_3_T, 2.5 (Q1–Q3 = 1.8–4.5) vs. O_3_T, 7 (Q1–Q3 = 1.5–10.3), *p* = 0.204. Depression assessed by HADS-D did not show significant differences between the patients without or with antidepressant treatment: (i) before O_3_T, 3 (Q1–Q3 = 0.8–9.3) vs. 5 (Q1–Q3 = 2.5–9), *p* = 0.688 or (ii) after O_3_T, 3 (Q1–Q3 = 0.8–5.3) vs. O_3_T, 4 (Q1–Q3 = 0–6.3), *p* = 0.826.

**Figure 1:**
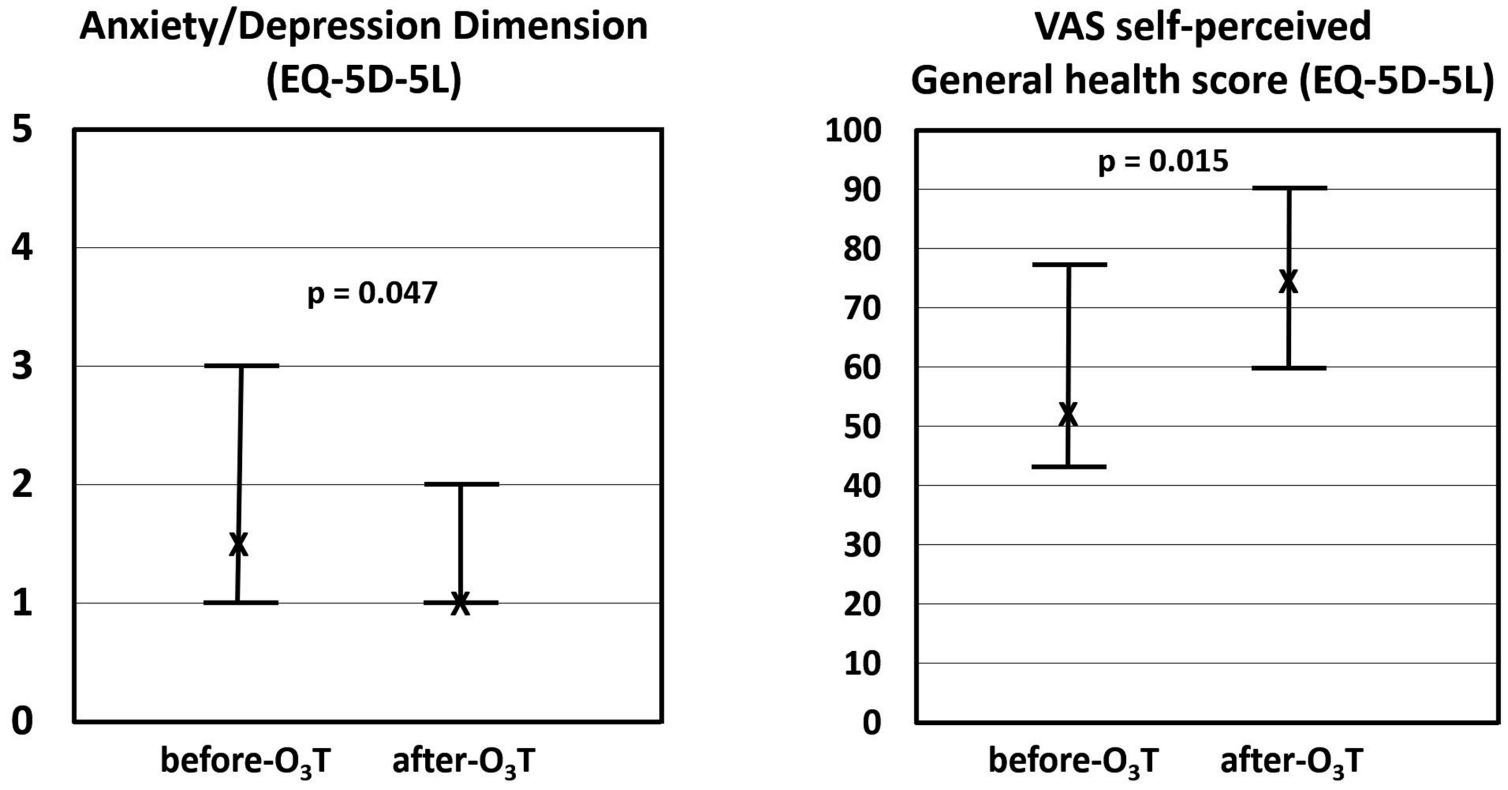
Hospital anxiety and depression scale (HADS). After ozone therapy (O_3_T), we found a significant decrease in: (i) HADS-Anxiety (HADS-A) from 6.5 (Q1–Q3 = 3–10.8) to 3.5 (Q1-Q = 2–6.8), *p* = 0.003 and (ii) HADS-Depression (HADS-D) from 4 (Q1–Q3 = 1.3–9) to 3.5 (Q1–Q3 = 0.3–5) after O3T (*p* = 0.022). X: median value. Bars: interquartile range.

Overall, the median HADS-A score was 6.5 (Q1–Q3 = 3–10.8) before O_3_T and 3.5 (Q1-Q = 2–6.8) after O_3_T (*p* = 0.003). The median HADS-D was 4 (Q1–Q3 = 1.3–9) before O_3_T and 3.5 (Q1–Q3 = 0.3–5) after O_3_T (*p* = 0.022) (Figure 1). In the group of 13 cancer patients: (i) the median HADS-A was 5 (Q1–Q3: 3–10.5) before O_3_T and 3 (Q1–Q3: 1.6–6.5) after O_3_T (*p* = 0.016); and (ii) the median HADS-D was 4 (Q1–Q3: 1–9) before O_3_T and 3 (Q1–Q3: 0–4.5) after O_3_T (*p* = 0.070).

The HADS-A results showed mild or higher anxiety levels (values ≥8) in eight (50%) patients before O_3_T and in three (18.8%) patients after O_3_T, (*p* = 0.063). The HADS-D results showed mild or higher depression levels in six patients (38%) before O_3_T and two patients (13%) after O_3_T (*p* = 0.125).

**Figure 2:**
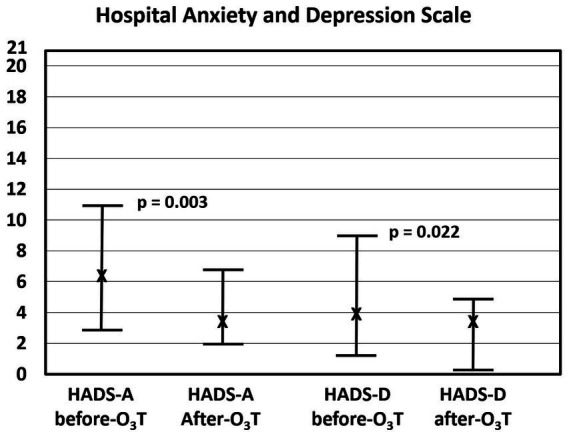
Assessment with the EQ-5D-5L questionnaire. (Left) Results on the anxiety/depression dimensions of the EQ-5D-5L significantly decreased after ozone therapy (O_3_T) from 1.5 (Q1–Q3: 1–3) to 1 (Q1–Q3: 1–2), *p* = 0.047. (Right) Visual analog scale results of self-perceived general health score significantly increased after O_3_T from 52.5 (Q1–Q3: 43–78) to 75 (Q1–Q3: 60–90), *p* = 0.015. X: median value. Bars: interquartile range.

After O_3_T, the EQ-5D-5L questionnaire showed: *(i) a significant improvement (decreased values) in the anxiety/depression dimension, from a median value of 1.5 (Q1–Q3: 1–3) to 1 (Q1–Q3: 1–2), *p* = 0.047; and (ii) a significant improvement (increased values) in the EQ VAS, from a median value of 52.5 (Q1–Q3: 43–78) to 75 (Q1–Q3: 60–90), *p* = 0.015 (Figure 2). In the group of 13 cancer patients, the EQ-5D-5L questionnaire showed changes in: the anxiety/depression dimension from a median value of 1 (Q1–Q3: 1–3) to 1 (Q1–Q3: 1–2), *p* = 0.188; and (ii) the EQ VAS, from a median value of 65 (Q1–Q3: 45–87.5) to 75 (Q1–Q3: 62.5–90), *p* = 0.033.

Before O_3_T, the HADS-A results showed a strong correlation with the anxiety/depression dimension of the EQ-5D-5L questionnaire (rho = 0.866, *p* < 0.001) and an inverse correlation with the EQ VAS (rho = −0.554, *p* = 0.026). Additionally, the HADS-D subscale results significantly correlated with age (rho = 0.521, *p* = 0.039) and the anxiety/depression dimension of the EQ-5D-5L (rho = 0.852, *p* < 0.001), and inversely with the EQ VAS (rho = −0.644, *p* = 0.007).

After O_3_T, the HADS-A results correlated with the anxiety/depression dimension of the EQ-5D-5L questionnaire (rho = 0.874, *p* < 0.001) and inversely with the EQ VAS (rho = −0.627, *p* = 0.009). The HADS-D results correlated with the anxiety/depression dimensions of the EQ-5D-5L (rho = 0.673, *p* = 0.004), and inversely with the EQ VAS (rho = −0.812, p < 0.001).

Table 2 shows a summary of the main results of the study group.

**Table 2:** Summary of the main results of the study group.

	Before-O_3_T median (Q1–Q3)	After-O_3_T median (Q1–Q3)	*p*-value
HADS-A (from 0 to 21)	6.5 (3–10.8)	3.5 (2–6.8)	0.003
HADS-D (from 0 to 21)	4 (1.3–9)	3.5 (0.3–5)	0.022
EQ-5D-5L: Anxiety/depression (from 1 to 5)	1.5 (1–3)	1 (1–2)	0.047
EQ-5D-5L: EQ VAS (from 0 to 100)	52.5 (43–78)	75 (60–90)	0.015

	Before-O_3_T Correlations (rho)	After-O_3_T Correlations (rho)	*p*-value before/after
HADS-A-EQ-5D-5L Anxiety/Depression-EQ-5D-5L, EQ VAS	0.866, –0.554	0.874, –0.627	< 0.001/< 0.001 0.026/0.009
HADS-D-EQ-5D-5L Anxiety/Depression-EQ-5D-5L, EQ VAS	0.852, –0.644	0.673, –0.812	< 0.001/0.004 0.007/<0.001

O_3_T, ozone therapy; Q1–Q3: quartiles 1 and 3; HADS, Hospital Anxiety and Depression Scale; HADS-A, HADS-Anxiety; HADS-D, HADS-Depression; EQ VAS, EQ-5D-5L Visual Analog Scale self-perceived general health status.

## 4. Discussion

Anxiety and depression are frequent disorders experienced by patients with severe disease, especially in those with chronic or refractory symptoms, producing a relevant impact on their HRQOL. In our study, adjuvant treatment with O_3_T of chronic and refractory symptoms in cancer survivors and patients with advanced diseases was associated with improvement in anxiety and depression, assessed by the specific HADS subscales.

In a recent study, we found that O_3_T could improve HRQOL using the EQ-5D-5L questionnaire (Clavo et al., 2023). However, the EQ-5D-5L questionnaire assesses the anxiety/depression dimension with only one question and it seemed justified to evaluate this dimension with a specific questionnaire focused on anxiety and depression. We used the Spanish version of the HADS, which is frequently used in our Chronic Pain Unit. In patients with different diagnoses, the Spanish HADS version has showed: (i) high test–retest reliability, with correlation coefficients above 0.85; (ii) high internal consistency, with a Cronbach’s alpha of 0.85 for anxiety and above 0.84 for depression; and (iii) high concurrent validity, with the Beck Depression Inventory and State–Trait Anxiety Inventory and with the mental domains of the Short-Form Health Survey (Herrero et al., 2003; Quintana et al., 2003).

The prevalence of depression is approximately 20% in cancer survivors, although the prevalence of self-reported depression using the HADS-D subscale seems to be lower, at approximately 13% (Boyes et al., 2013). We previously described that 34.9% of patients submitted to our Chronic Pain Unit had been prescribed anxiolytic or antidepressants treatment. Of them, 24.2% were on anxiolytics only, 40.2% were on antidepressants only, and 35.5% were on both treatments. In that review, no patient had an antidepressant prescription for pain management (usually duloxetine or amitriptyline) (Caramés et al., 2021). In our study group, before O_3_T, nine patients (56%) were on anxiolytic (38%) or antidepressant (38%) treatment, and three (19%) were on treatment for both disorders.

The treatment of anxiety and depression in cancer survivors and in patients with refractory symptoms of advanced diseases is similar to that of different patient populations, including pharmacologic treatment and cognitive behavioral therapy. However, the patient fears the formal diagnosis of anxiety and depression due to the social stigma surrounding mental illness. So, patients are hesitant to express a desire for psychiatric evaluation and consider that treatment is not essential or a priority (Kim et al., 2015). As such, in the EU, most patients reported neither provider discussions nor the use of professional psychosocial counseling or support groups (Forsythe et al., 2013). Additionally, anxiety and depression were not fully controlled in our group of patients, and these symptoms remained despite half of the patients already being on anxiolytic and/or antidepressant treatment.

Affective disorders and depression are associated with high levels of oxidative stress markers [such as 8-hydroxydeoxyguanosine (8-OHdG)], proinflammatory cytokines (such as interleukins (IL) IL-1 and IL-6 and tumor necrosis factor alpha (TNFα)), as well as with decreased levels of antioxidants (such as coenzyme Q10, glutathione peroxidase, and zinc) (Ng et al., 2008; Maes et al., 2011; Leonard and Maes, 2012; Lindqvist et al., 2017). Additionally, oxidative stress is associated with low brain-derived neurotrophic factor (BDNF) and subsequent decreases in Nrf2 activity (Bouvier et al., 2017). Clinically, in comparison with healthy controls, patients with major depressive disorder have significantly higher levels of proinflammatory cytokines such as IL-6, TNFα, 8-OHdG, and F2-isoprostanes. Furthermore, those parameters are associated with the success of antidepressant treatment. Over the course of treatment, patients without response to selective serotonin reuptake inhibitors (SSRIs) showed an increase in 8-OHdG levels, whereas patients showing a response to SSRIs showed a decrease in IL-6 levels (Lindqvist et al., 2017).

Several studies on the impact of oxidative stress on brain function have been based on experimental models of ozone “inhalation,” which described changes in brain neurotransmitters and increases in dopamine and noradrenaline (Gonzalez-Pina and Paz, 1997), oxidized dopamine species (Santiago-Lopez et al., 2010), memory alterations (Avila-Costa et al., 1999; Mokoena et al., 2015), anxiety, and depression, even with antidepressant treatment (Mokoena et al., 2015). Clinically, a detrimental effect of environmental ozone exposure has also been associated with increased psychiatric emergency services admissions (Bernardini et al., 2019), depression (Kioumourtzoglou et al., 2017), and other psychiatric disorders (Gladka et al., 2018). However, some systematic reviews with meta-analyses did not find a statistically significant association between the role of ozone and depression (Zhao et al., 2018; Fan et al., 2020; Borroni et al., 2022). Further clinical studies in this field are required. Notably, we highlight that medical ozone administration must specifically avoid the inhalation of ozone, so, these studies would not apply to O_3_T.

Conversely, clinical O_3_T is based on appropriate: (i) routes of ozone administration (inhalation must be avoided) and (ii) ozone concentrations. We previously described the relationship between oxidative stress and inflammation and the toxicity of chemotherapy in detail (Clavo et al., 2021, 2022). When low/moderate ozone concentrations are properly administered by systemic routes, a relevant part of ozone will be removed by the antioxidant defenses of the medium (rectal mucosa when rectal insufflation is used or the blood when autohemotherapy is used). The remaining ozone will interact with biomolecules as polyunsaturated fatty acids from cell membranes or blood cells, or other components of rectal mucosa or plasma, to generate reactive species of oxygen, second messengers and lipid peroxides, which can reach distant tissues. This way, O_3_T produces a controlled and transient oxidative stress that indirectly will induce an adaptive response of the organism, with overregulation of Nrf2 (which leads to potentiation of antioxidant systems) and downregulation of NF-kβ and proinflammatory cytokines (Re et al., 2014; Bocci and Valacchi, 2015; Galie et al., 2019; Viebahn-Haensler and Leon Fernandez, 2021). These actions are in the opposite direction that those involved in the production of anxiety and depression that were described in the two previous paragraphs about “ozone inhalation.” Thus, the enhancement in Nrf2 levels and antioxidant systems by O_3_T may be of interest in the management of anxiety or depression, as supported by the described (i) antioxidant properties for some antidepressants such as desvenlafaxine, desipramine, and venlafaxine (Gaur and Kumar, 2010; Silva et al., 2016); (ii) decreased oxidative stress damage induced by chronic mild stress by the modulation of Nrf2 in the prefrontal cortex by antidepressants (Martin-Hernandez et al., 2016); and (iii) beneficial effect of O_3_T (avoiding inhalation) in experimental models on aging and neurodegenerative disorders (El-Mehi and Faried, 2020; Scassellati et al., 2020) and a clinical study on depression in older patients (Coppola et al., 2010).

After O_3_T, the patients in our study showed a decrease in anxiety and depression in (i) the anxiety/depression dimension of the EQ-5D-5L questionnaire and (ii) the HADS-A and HADS-D subscales. Both HADS subscales showed: (i) a marked correlation with the anxiety/depression dimension of the EQ-5D-5L questionnaire, and (ii) the correlation was higher with anxiety than with depression. These results in our study agree with a previous larger study with 245 patients comparing the same anxiety/depression dimension of the EQ-5D-3L questionnaire with the HADS subscales (Thayabaranathan et al., 2022). HADS-A and HADS-D subscales also showed a significant correlation with the VAS self-perceived general health status. However, here, the correlation was higher with depression than with anxiety. These results agree with the findings of studies showing that a high prevalence of anxiety and depression predicts a low HRQOL in cancer patients, but also that depression has a more pervasive association with multiple other domains of HRQL (Brown et al., 2010). On the other hand, our results with O_3_T in patients with refractory symptoms of cancer treatment and advanced nononcologic diseases, agree with the results of O_3_T on anxiety and depression in three previous reports using systemic O_3_T in different kinds of patients: (i) in older patients with mild to moderate depression and mild cognitive impairment, assessed by the Hamilton and Montgomery scales (Coppola et al., 2010), in patients with fibromyalgia assessed by the Beck Depression Inventory and the State and Trait Anxiety Inventory (Hidalgo-Tallon et al., 2013), and (ii) in patients with insomnia and coronary heart disease, assessed by the HADS questionnaire (Li et al., 2021).

Among the limitations of this study, we highlight the following: (i) This study had a small sample size. Currently, the assessment of anxiety and depression is a specific aim of our larger ongoing studies with O_3_T in cancer (NCT04299893) and noncancer (NCT05417737) patients. (ii) The improvement in anxiety and depression could have been partially related to the improvement in physical symptoms in most patients, especially in the three patients only treated with topical O_3_ administration. (iii) This is a nonrandomized clinical trial, so a potential placebo effect could not be completely ruled out, including the potential effect on anxiety and depression of closer follow-up during the ozone treatment period. However, two relevant aspects should be considered regarding the potential placebo effect in points (ii) and (iii): (a) patients in this study suffered advanced disease and chronic symptoms for many months before O_3_T; (b) as mentioned above, the role of oxidative stress in anxiety and depression has been well-described (Ng et al., 2008; Maes et al., 2011; Leonard and Maes, 2012; Lindqvist et al., 2017). So, the well-described effect of O_3_T in modulating oxidative stress and inflammation overall (Re et al., 2014; Bocci and Valacchi, 2015; Galie et al., 2019; Viebahn-Haensler and Leon Fernandez, 2021) and at the brain level (Coppola et al., 2010; El-Mehi and Faried, 2020; Scassellati et al., 2020) probably played a direct role in our patients, especially in those treated with systemic O_3_T (most of them). Finally, we would like to mention two additional limitations: (iv) the study was focused on patients with severe diseases treated with O_3_T because of refractory symptoms, but they were treated different cancer-related and noncancer-related symptoms and diagnosis, and (v) anxiety, depression, or treatment for anxiety and depression were not present in all patients. Results could be conditioned by these clinical parameters. Further specifically addressed studies are required, and our ongoing studies will more thoroughly assess the role of O_3_T in anxiety and depression.

### 4.1. Conclusions

In this preliminary study, using the Hospital Anxiety and Depression Scale questionnaire, patients with refractory symptoms of cancer treatment and advanced disease showed decreased mild or higher levels of both anxiety and depression after ozone therapy. The effect of ozone therapy on the psychological field merits focused research, and related studies are ongoing.

## Data availability statement

The raw data supporting the conclusions of this article will be made available by the authors, without undue reservation.

## Ethics statement

The studies involving human participants were reviewed and approved by Provincial Research Ethics Committee of Las Palmas, Spain. The patients/participants provided their written informed consent to participate in this study.

## Author contributions

BC, DR-A, JD-G, PS-A, and FR-E: conceptualization. BC, YR-F, and PS-A: formal analysis. BC, YR-F, PS-A, and FR-E: methodology. DR-A, MF, and SG: initial management and oncology follow-up. BC, AC-M, CG-L, DG-B, and MC: treatment with ozone therapy. JD-G, SC, HL, and JH-F: evaluation of HADS questionnaire. BC, JD-G, YR-F, HL, JH-F, PS-A, and FR-E: writing—original draft. BC, AC-M, JD-G, SC, YR-F, HL, MF, DR-A, SG, CG-L, DG-B, MC, JH-F, PS-A, and FR-E: writing—review and editing and approval of the final version. BC and DR-A: funding acquisition. All authors have read and agreed to the published version of the manuscript.

## Funding

This study was partially supported by a grant (PI 19/00458) from the Instituto de Salud Carlos III (Spanish Ministry of Science and Innovation, Madrid, Spain, and European Regional Development Fund—ERDF); a grant (PI 016/2019) from the Fundación DISA (Las Palmas, Spain); a grant (BF1-19-13) from the Fundación Española del Dolor (Spanish Pain Foundation, Madrid, Spain); a grant I42/20 from the Ilustre Colegio Oficial de Médicos de Las Palmas, Spain; a grant (CIGC2021) from the Cabildo de Gran Canaria, Las Palmas, Spain; and a grant (ENF22/10) from the Fundación Canaria Instituto Investigación Sanitaria de Canarias (FIISC), Las Palmas, Spain. The use of the other ozone therapy device in this study (Ozonobaric-P, SEDECAL, Madrid, Spain) was supported by a grant (COV20/00702) from the Instituto de Salud Carlos III (Spanish Ministry of Science and Innovation, Madrid, Spain).

## Conflict of interest

The authors declare that the research was conducted in the absence of any commercial or financial relationships that could be construed as a potential conflict of interest.

## Publisher’s note

All claims expressed in this article are solely those of the authors and do not necessarily represent those of their affiliated organizations, or those of the publisher, the editors and the reviewers. Any product that may be evaluated in this article, or claim that may be made by its manufacturer, is not guaranteed or endorsed by the publisher.

## References

[ref1] Avila-Costa M. R. Colin-Barenque L. Fortoul T. I. Machado-Salas P. Espinosa-Villanueva J. Rugerio-Vargas C.. (1999). Memory deterioration in an oxidative stress model and its correlation with cytological changes on rat hippocampus CA1. Neurosci. Lett. 270, 107–109. doi: 10.1016/S0304-3940(99)00458-9, PMID: 10462109

[ref2] Bernardini F. Attademo L. Trezzi R. Gobbicchi C. Balducci P. M. Del Bello V.. (2019). Air pollutants and daily number of admissions to psychiatric emergency services: evidence for detrimental mental health effects of ozone. Epidemiol. Psychiatr. Sci. 29:e66. doi: 10.1017/S2045796019000623, PMID: 31690359 PMC8061137

[ref3] Bocci V. Valacchi G. (2015). Nrf2 activation as target to implement therapeutic treatments. Front. Chem. 3:4. doi: 10.3389/fchem.2015.00004, PMID: 25699252 PMC4313773

[ref4] Borroni E. Pesatori A. C. Bollati V. Buoli M. Carugno M. (2022). Air pollution exposure and depression: a comprehensive updated systematic review and meta-analysis. Environ. Pollut. 292:118245. doi: 10.1016/j.envpol.2021.118245, PMID: 34600062

[ref5] Bouvier E. Brouillard F. Molet J. Claverie D. Cabungcal J. H. Cresto N.. (2017). Nrf2-dependent persistent oxidative stress results in stress-induced vulnerability to depression. Mol. Psychiatry 22:1795. doi: 10.1038/mp.2016.211, PMID: 27801891 PMC8127815

[ref6] Boyes A. W. Girgis A. D'Este C. A. Zucca A. C. Lecathelinais C. Carey M. L. (2013). Prevalence and predictors of the short-term trajectory of anxiety and depression in the first year after a cancer diagnosis: a population-based longitudinal study. J. Clin. Oncol. 31, 2724–2729. doi: 10.1200/JCO.2012.44.7540, PMID: 23775970

[ref7] Brown L. F. Kroenke K. Theobald D. E. Wu J. Tu W. (2010). The association of depression and anxiety with health-related quality of life in cancer patients with depression and/or pain. Psychooncology 19, 734–741. doi: 10.1002/pon.1627, PMID: 19777535 PMC2888919

[ref8] Caramés M. A. Navarro M. Pérez-Lehmann C. Hernández-Rodríguez J. Lázaro-Archilla J. Clavo B. (2021). Descriptive study on the profile of patients sent to the chronic pain treatment unit of the University Hospital of Gran Canaria Dr. Negrín (UTDC-HUGCDN) as a basis for the reorganization of this. [Estudio descriptivo sobre el perfil de los pacientes derivados a la Unidad de Tratamiento del dolor Crónico del Hospital Universitario de gran Canaria Dr. Negrín (UTDC-HUGCDN) Como base Para la reorganización de esta]. Rev. Soc. Esp. Dol.or 25, 254–263. doi: 10.20986/resed.2021.3939/2021

[ref9] Clavo B. Canovas-Molina A. Ramallo-Farina Y. Federico M. Rodriguez-Abreu D. Galvan S.. (2023). Effects of ozone treatment on health-related quality of life and toxicity induced by radiotherapy and chemotherapy in symptomatic Cancer survivors. Int. J. Environ. Res. Public Health 20:1479. doi: 10.3390/ijerph20021479, PMID: 36674232 PMC9859304

[ref10] Clavo B. Ceballos D. Gutierrez D. Rovira G. Suarez G. Lopez L.. (2013). Long-term control of refractory hemorrhagic radiation proctitis with ozone therapy. J. Pain Symptom Manag. 46, 106–112. doi: 10.1016/j.jpainsymman.2012.06.017, PMID: 23102757

[ref11] Clavo B. Gutierrez D. Martin D. Suarez G. Hernandez M. A. Robaina F. (2005). Intravesical ozone therapy for progressive radiation-induced Hematuria. J. Altern. Complement. Med. 11, 539–541. doi: 10.1089/acm.2005.11.539, PMID: 15992242

[ref12] Clavo B. Martinez-Sanchez G. Rodriguez-Esparragon F. Rodriguez-Abreu D. Galvan S. Aguiar-Bujanda D.. (2021). Modulation by ozone therapy of oxidative stress in chemotherapy-induced peripheral neuropathy: the background for a randomized clinical trial. Int. J. Mol. Sci. 22:2802. doi: 10.3390/ijms22062802, PMID: 33802143 PMC7998838

[ref13] Clavo B. Navarro M. Federico M. Borrelli E. Jorge I. J. Ribeiro I.. (2021). Long-term results with adjuvant ozone therapy in the Management of Chronic Pelvic Pain Secondary to Cancer treatment. Pain Med. 22, 2138–2141. doi: 10.1093/pm/pnaa459, PMID: 33738491 PMC8557383

[ref14] Clavo B. Rodríguez-Abreu D. Galván S. Federico M. Martínez-Sánchez G. Ramallo-Fariña Y.. (2022). Long-term improvement by ozone treatment in chronic pain secondary to chemotherapy-induced peripheral neuropathy: a preliminary report. Front. Physiol. 13:935269. doi: 10.3389/fphys.2022.935269, PMID: 36111149 PMC9468657

[ref15] Clavo B. Santana-Rodriguez N. Gutierrez D. Lopez J. C. Suarez G. Lopez L.. (2013). Long-term improvement in refractory headache following ozone therapy. J. Altern. Complement. Med. 19, 453–458. doi: 10.1089/acm.2012.0273, PMID: 23215625

[ref16] Clavo B. Santana-Rodriguez N. Llontop P. Gutierrez D. Ceballos D. Mendez C.. (2015). Ozone therapy in the Management of Persistent Radiation-Induced Rectal Bleeding in prostate Cancer patients. Evid. Based Complement. Alternat. Med. 2015:480369. doi: 10.1155/2015/480369, PMID: 26357522 PMC4556325

[ref17] Clavo B. Suarez G. Aguilar Y. Gutierrez D. Ponce P. Cubero A.. (2011). Brain ischemia and hypometabolism treated by ozone therapy. Forsch. Komplementmed. 18, 283–287. doi: 10.1159/000333795, PMID: 22105041

[ref18] Coppola L. Luongo C. Pastore A. Masciello C. Parascandola R. R. Mastrolorenzo L.. (2010). Ozonized autohaemotransfusion could be a potential rapid-acting antidepressant medication in elderly patients. Int. J. Geriatr. Psychiatry 25, 208–213. doi: 10.1002/gps.2322, PMID: 19521954

[ref19] DeJean D. Giacomini M. Vanstone M. Brundisini F. (2013). Patient experiences of depression and anxiety with chronic disease: a systematic review and qualitative meta-synthesis. Ont. Health Technol. Assess. Ser. 13, 1–33. PMID: 24228079 PMC3817854

[ref20] El-Mehi A. E. Faried M. A. (2020). Controlled ozone therapy modulates the neurodegenerative changes in the frontal cortex of the aged albino rat. Ann. Anat. 227:151428. doi: 10.1016/j.aanat.2019.151428 31610254

[ref21] European Commission (2000). European Comission: Estimates of survival, by country and cancer site, 2000–2007. Available at: https://ecis.jrc.ec.europa.eu/explorer.php?$0-2$1-AEE$2-All$4-1,2$3-0$6-0,14$5-2000,2007$7-1$CRelativeSurvivalAgeGroup$X0_14-$X0_15-RSC$CRelativeSurvivalFollow$X1_14-$X1_-1-$X1_15-RSC.In (accessed February 14th, 2023).

[ref22] European Commission (2023). Persons reporting a chronic disease, by disease, sex, age and educational attainment level. Available at: https://ec.europa.eu/eurostat/databrowser/view/HLTH_EHIS_CD1E/bookmark/table?lang=en&bookmarkId=2d249b06-f173-48b3-b6ed-e90b57e6f683&page=time:2019 (accessed February 14th, 2023),

[ref23] Fan S. J. Heinrich J. Bloom M. S. Zhao T. Y. Shi T. X. Feng W. R.. (2020). Ambient air pollution and depression: a systematic review with meta-analysis up to 2019. Sci. Total Environ. 701:134721. doi: 10.1016/j.scitotenv.2019.134721 31715478

[ref24] Forsythe L. P. Kent E. E. Weaver K. E. Buchanan N. Hawkins N. A. Rodriguez J. L.. (2013). Receipt of psychosocial care among cancer survivors in the United States. J. Clin. Oncol. 31, 1961–1969. doi: 10.1200/JCO.2012.46.2101, PMID: 23610114 PMC3661934

[ref25] Galie M. Covi V. Tabaracci G. Malatesta M. (2019). The role of Nrf2 in the antioxidant cellular response to medical ozone exposure. Int. J. Mol. Sci. 20:4009. doi: 10.3390/ijms20164009, PMID: 31426459 PMC6720777

[ref26] Gaur V. Kumar A. (2010). Protective effect of desipramine, venlafaxine and trazodone against experimental animal model of transient global ischemia: possible involvement of NO-cGMP pathway. Brain Res. 1353, 204–212. doi: 10.1016/j.brainres.2010.07.004, PMID: 20624374

[ref27] Gladka A. Rymaszewska J. Zatonski T. (2018). Impact of air pollution on depression and suicide. Int. J. Occup. Med. Environ. Health 31, 711–721. doi: 10.13075/ijomeh.1896.01277, PMID: 30281038

[ref28] Gonzalez-Pina R. Paz C. (1997). Brain monoamine changes in rats after short periods of ozone exposure. Neurochem. Res. 22, 63–66. doi: 10.1023/A:1027329405112, PMID: 9021764

[ref29] Gotze H. Friedrich M. Taubenheim S. Dietz A. Lordick F. Mehnert A. (2020). Depression and anxiety in long-term survivors 5 and 10 years after cancer diagnosis. Support Care Cancer 28, 211–220. doi: 10.1007/s00520-019-04805-1, PMID: 31001695

[ref30] Greer J. A. Solis J. M. Temel J. S. Lennes I. T. Prigerson H. G. Maciejewski P. K.. (2011). Anxiety disorders in long-term survivors of adult cancers. Psychosomatics 52, 417–423. doi: 10.1016/j.psym.2011.01.014, PMID: 21907059 PMC3172571

[ref31] Herdman M. Gudex C. Lloyd A. Janssen M. Kind P. Parkin D.. (2011). Development and preliminary testing of the new five-level version of EQ-5D (EQ-5D-5L). Qual. Life Res. 20, 1727–1736. doi: 10.1007/s11136-011-9903-x, PMID: 21479777 PMC3220807

[ref32] Hernandez G. Garin O. Pardo Y. Vilagut G. Pont A. Suarez M.. (2018). Validity of the EQ-5D-5L and reference norms for the Spanish population. Qual. Life Res. 27, 2337–2348. doi: 10.1007/s11136-018-1877-5, PMID: 29767329

[ref33] Herrero M. J. Blanch J. Peri J. M. De Pablo J. Pintor L. Bulbena A. (2003). A validation study of the hospital anxiety and depression scale (HADS) in a Spanish population. Gen. Hosp. Psychiatry 25, 277–283. doi: 10.1016/S0163-8343(03)00043-4, PMID: 12850660

[ref34] Hidalgo-Tallon J. Menendez-Cepero S. Vilchez J. S. Rodriguez-Lopez C. M. Calandre E. P. (2013). Ozone therapy as add-on treatment in fibromyalgia management by rectal insufflation: an open-label pilot study. J. Altern. Complement. Med. 19, 238–242. doi: 10.1089/acm.2011.0739, PMID: 23046293

[ref35] Ji X. Cummings J. R. Gilleland Marchak J. Han X. Mertens A. C. (2020). Mental health among nonelderly adult cancer survivors: a national estimate. Cancer 126, 3768–3776. doi: 10.1002/cncr.32988, PMID: 32538481

[ref36] Kim J. L. Cho J. Park S. Park E. C. (2015). Depression symptom and professional mental health service use. BMC Psychiatry 15:261. doi: 10.1186/s12888-015-0646-z 26497588 PMC4619991

[ref37] Kioumourtzoglou M. A. Power M. C. Hart J. E. Okereke O. I. Coull B. A. Laden F.. (2017). The association between air pollution and onset of depression among middle-aged and older women. Am. J. Epidemiol. 185, 801–809. doi: 10.1093/aje/kww163, PMID: 28369173 PMC5411676

[ref38] Leonard B. Maes M. (2012). Mechanistic explanations how cell-mediated immune activation, inflammation and oxidative and nitrosative stress pathways and their sequels and concomitants play a role in the pathophysiology of unipolar depression. Neurosci. Biobehav. Rev. 36, 764–785. doi: 10.1016/j.neubiorev.2011.12.005, PMID: 22197082

[ref39] Li Y. Feng X. Ren H. Huang H. Wang Y. Yu S. (2021). Low-dose ozone therapy improves sleep quality in patients with insomnia and coronary heart disease by elevating serum BDNF and GABA. Bull. Exp. Biol. Med. 170, 493–498. doi: 10.1007/s10517-021-05095-6, PMID: 33713235

[ref40] Li H. Ge S. Greene B. Dunbar-Jacob J. (2019). Depression in the context of chronic diseases in the United States and China. Int. J. Nurs. Sci. 6, 117–122. doi: 10.1016/j.ijnss.2018.11.007, PMID: 31406877 PMC6608796

[ref41] Lindqvist D. Dhabhar F. S. James S. J. Hough C. M. Jain F. A. Bersani F. S.. (2017). Oxidative stress, inflammation and treatment response in major depression. Psychoneuroendocrinology 76, 197–205. doi: 10.1016/j.psyneuen.2016.11.031, PMID: 27960139 PMC5272818

[ref42] Lloyd S. Baraghoshi D. Tao R. Garrido-Laguna I. Gilcrease G. W.. (2019). Mental health disorders are more common in colorectal Cancer survivors and associated with decreased overall survival. Am. J. Clin. Oncol. 42, 355–362. doi: 10.1097/COC.0000000000000529, PMID: 30844850 PMC6433523

[ref43] Maes M. Galecki P. Chang Y. S. Berk M. (2011). A review on the oxidative and nitrosative stress (O&NS) pathways in major depression and their possible contribution to the (neuro)degenerative processes in that illness. Prog. Neuro-Psychopharmacol. Biol. Psychiatry 35, 676–692. doi: 10.1016/j.pnpbp.2010.05.004 20471444

[ref44] Martin-Hernandez D. Bris A. G. MacDowell K. S. Garcia-Bueno B. Madrigal J. L. Leza J. C.. (2016). Modulation of the antioxidant nuclear factor (erythroid 2-derived)-like 2 pathway by antidepressants in rats. Neuropharmacology 103, 79–91. doi: 10.1016/j.neuropharm.2015.11.029, PMID: 26686388

[ref45] Mitchell A. J. Meader N. Symonds P. (2010). Diagnostic validity of the hospital anxiety and depression scale (HADS) in cancer and palliative settings: a meta-analysis. J. Affect. Disord. 126, 335–348. doi: 10.1016/j.jad.2010.01.067, PMID: 20207007

[ref46] Mokoena M. L. Harvey B. H. Viljoen F. Ellis S. M. Brink C. B. (2015). Ozone exposure of Flinders sensitive line rats is a rodent translational model of neurobiological oxidative stress with relevance for depression and antidepressant response. Psychopharmacology 232, 2921–2938. doi: 10.1007/s00213-015-3928-8, PMID: 25877744

[ref47] Ng F. Berk M. Dean O. Bush A. I. (2008). Oxidative stress in psychiatric disorders: evidence base and therapeutic implications. Int. J. Neuropsychopharmacol. 11, 851–876. doi: 10.1017/S1461145707008401, PMID: 18205981

[ref48] Quintana J. M. Padierna A. Esteban C. Arostegui I. Bilbao A. Ruiz I. (2003). Evaluation of the psychometric characteristics of the Spanish version of the hospital anxiety and depression scale. Acta Psychiatr. Scand. 107, 216–221. doi: 10.1034/j.1600-0447.2003.00062.x, PMID: 12580829

[ref49] Rabin R. Gudex C. Selai C. Herdman M. (2014). From translation to version management: a history and review of methods for the cultural adaptation of the EuroQol five-dimensional questionnaire. Value Health 17, 70–76. doi: 10.1016/j.jval.2013.10.006, PMID: 24438719

[ref50] Ramos-Goni J. M. Craig B. M. Oppe M. Ramallo-Farina Y. Pinto-Prades J. L. Luo N.. (2018). Handling data quality issues to estimate the Spanish EQ-5D-5L value set using a hybrid interval regression approach. Value Health 21, 596–604. doi: 10.1016/j.jval.2017.10.023, PMID: 29753358

[ref51] Re L. Martinez-Sanchez G. Bordicchia M. Malcangi G. Pocognoli A. Morales-Segura M. A.. (2014). Is ozone pre-conditioning effect linked to Nrf2/EpRE activation pathway *in vivo*? A preliminary result. Eur. J. Pharmacol. 742, 158–162. doi: 10.1016/j.ejphar.2014.08.029, PMID: 25218903

[ref52] Santiago-Lopez D. Bautista-Martinez J. A. Reyes-Hernandez C. I. Aguilar-Martinez M. Rivas-Arancibia S. (2010). Oxidative stress, progressive damage in the substantia nigra and plasma dopamine oxidation, in rats chronically exposed to ozone. Toxicol. Lett. 197, 193–200. doi: 10.1016/j.toxlet.2010.05.020, PMID: 20541596

[ref53] Scassellati C. Galoforo A. C. Bonvicini C. Esposito C. Ricevuti G. (2020). Ozone: a natural bioactive molecule with antioxidant property as potential new strategy in aging and in neurodegenerative disorders. Ageing Res. Rev. 63:101138. doi: 10.1016/j.arr.2020.101138, PMID: 32810649 PMC7428719

[ref54] Silva M. C. de Sousa C. N. Gomes P. X. de Oliveira G. V. Araujo F. Y. Ximenes N. C.. (2016). Evidence for protective effect of lipoic acid and desvenlafaxine on oxidative stress in a model depression in mice. Prog. Neuro-Psychopharmacol. Biol. Psychiatry 64, 142–148. doi: 10.1016/j.pnpbp.2015.08.002 26265141

[ref55] Thayabaranathan T. Andrew N. E. Stolwyk R. Lannin N. A. Cadilhac D. A. (2022). Comparing the EQ-5D-3L anxiety or depression domain to the hospital anxiety and depression scale to identify anxiety or depression after stroke. Top. Stroke Rehabil. 29, 146–155. doi: 10.1080/10749357.2021.1895494, PMID: 33726636

[ref56] Viebahn-Haensler R. Leon Fernandez O. S. (2021). Ozone in medicine. The low-dose ozone concept and its basic biochemical mechanisms of action in chronic inflammatory diseases. Int. J. Mol. Sci. 22:7890. doi: 10.3390/ijms22157890, PMID: 34360655 PMC8346137

[ref57] Waked I. S. Nagib S. H. Omar M. T. A. (2013). A single blinded randomized controlled clinical trial on the efficacy of ozone therapy on breast cancer-related lymphedema. Cancer Clin Oncol 2, 93–106. doi: 10.5539/cco.v2n2p93

[ref58] Zhao T. Markevych I. Romanos M. Nowak D. Heinrich J. (2018). Ambient ozone exposure and mental health: a systematic review of epidemiological studies. Environ. Res. 165, 459–472. doi: 10.1016/j.envres.2018.04.015, PMID: 29728258

[ref59] Zigmond A. S. Snaith R. P. (1983). The hospital anxiety and depression scale. Acta Psychiatr. Scand. 67, 361–370. doi: 10.1111/j.1600-0447.1983.tb09716.x, PMID: 6880820

